# Rethinking healthcare through aging biology

**DOI:** 10.18632/aging.206262

**Published:** 2025-05-29

**Authors:** Marco Demaria

**Affiliations:** 1 European Research Institute for the Biology of Ageing (ERIBA), University Medical Center Groningen (UMCG), University of Groningen (RUG), Netherlands

**Keywords:** healthcare, aging, senolytics, epigenetics, medical education

Modern medicine has revolutionized the way we diagnose and treat diseases, achieving remarkable success in extending life expectancy. Yet, despite these advances, the traditional disease-centric healthcare model has significant limitations. This approach typically kicks in only after pathology has manifested—when patients exhibit symptoms, seek treatment, receive a diagnosis, and begin therapy. While reactive care has its merits, it increasingly falls short in addressing the needs of aging populations. As people age, they often develop a constellation of chronic conditions—multimorbidity—that strains the healthcare system and diminishes quality of life. Conditions such as cardiovascular disease, type 2 diabetes, osteoarthritis, neurodegeneration, and cancer frequently coexist, leading to complex and often ineffective treatments. Polypharmacy—the use of multiple medications to treat co-existing diseases—introduces further complications, including drug interactions, side effects, reduced adherence to treatment regimens, and increased hospitalizations.

Moreover, this disease-specific focus neglects the underlying causes of age-related decline—the very mechanisms that fuel the development of these diseases. However, recent breakthroughs in aging research have unveiled an exciting opportunity: the shared biological roots of many age-related diseases. These mechanisms, known as the *hallmarks of aging* [1], often precede the onset of disease by decades. Targeting these aging processes before diseases fully develop offers a bold new approach: not just treating diseases, but preventing them in the first place.

This shift in focus from reactive disease management to proactive healthspan extension is transformative. By intervening early in the aging process, we could delay or even prevent multiple diseases, addressing not just the symptoms, but the biological declines that underlie them. In this context, cutting-edge interventions such as senolytics and rapalogs exemplify the promising potential of targeting aging itself. Senolytics, which selectively eliminate senescent cells that accumulate with age and contribute to chronic inflammation and tissue dysfunction, have shown promise in extending healthspan and alleviating a range of age-related conditions [2–4]. Likewise, rapalogs—drugs that target the mTOR pathway, a central regulator of cell growth and metabolism—have demonstrated the ability to extend lifespan and improve healthspan by promoting autophagy, enhancing immune function, and reducing inflammation [5, 6]. As clinical trials continue, these interventions are poised to transform aging medicine, but the road to widespread clinical application remains challenging.

Timing is crucial in this new paradigm. Evidence suggests that age-related damage begins in midlife, long before clinical disease manifests. Intervening during this critical window may significantly delay or reduce the impact of multimorbidity. However, some damage may be irreversible by midlife, requiring more aggressive interventions. This realization has given rise to an even more ambitious goal: preventing the initiation of aging mechanisms altogether. Unlike reparative strategies that address damage after it occurs, this preventative approach focuses on maintaining cellular and systemic homeostasis throughout life to avert molecular dysfunctions.

Lifestyle interventions remain the cornerstone of this prevention strategy. Regular physical activity, caloric restriction, metabolic flexibility, quality sleep, stress management, and avoidance of environmental toxins all influence the rate of biological aging. Emerging technologies, such as multi-omic profiling and biological age diagnostics, are allowing individuals to track their physiological trajectory and tailor lifestyle choices accordingly [7, 8]. Early life interventions are equally promising, with emerging evidence suggesting that optimizing maternal nutrition, minimizing early-life inflammation, and maintaining a healthy microbiome can set the stage for better long-term health outcomes.

This proactive approach not only holds promise for healthier aging, but also for more sustainable healthcare. The traditional disease-centric model incurs the highest costs, primarily due to late-stage treatments, chronic disease management, and the burden of polypharmacy. The second paradigm—targeting aging mechanisms after damage has occurred—offers the potential for reduced long-term costs by preventing disease progression and avoiding intensive care. However, the most cost- effective approach is the third model, which seeks to prevent the accumulation of aging-related damage entirely. By delaying or reducing the onset of chronic diseases, we can minimize the need for prolonged medical care and shift much of the burden away from hospitals and clinics to community-based or even home-based interventions. While upfront investments in research, early detection, and preventive care are necessary, the long-term savings—both economic and societal—could be profound.

Each of these paradigms requires distinct research priorities and funding strategies. The disease-centric model, for example, has long dominated biomedical research, driving advances in pathology-specific interventions. However, it has largely overlooked the interconnected biological mechanisms underlying multimorbidity. The second paradigm, focused on treating aging-related damage, calls for more translational and interdisciplinary research that bridges basic science with clinical applications. This includes the development of novel biomarkers for biological age and interventions that target shared molecular pathways. The third paradigm—preventing aging-related damage—demands a systemic shift toward predictive and preventative research, with an emphasis on multi-omic data, lifestyle interventions, and early-life interventions. Crucially, success in this third paradigm must be evaluated not by short-term clinical outcomes, but by its long-term impact on health and disease prevention (Figure 1).

**Figure 1:**
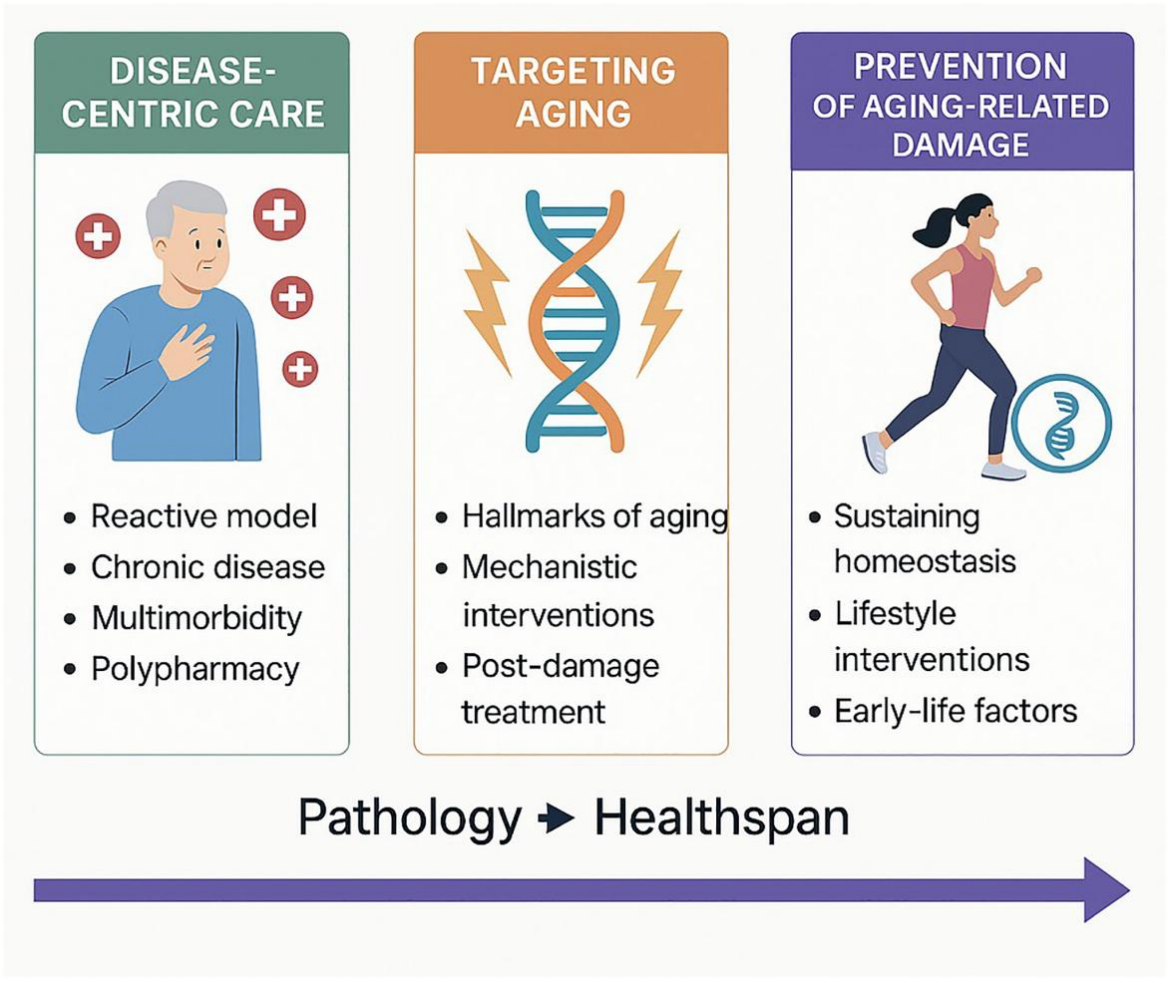
Shifting healthcare paradigms from disease management to proactive healthspan extension.

The transition to these new paradigms of healthcare will also require a profound transformation in medical education. Traditional training focuses on organ systems and specific diseases, preparing clinicians to diagnose and treat diseases individually. However, this model is ill-suited to managing the complexity of multimorbidity and aging-related decline. The second paradigm, targeting aging- related damage, necessitates an integrative curriculum that incorporates geroscience, systems biology, and translational medicine. The third paradigm, which prioritizes prevention, demands a radical shift in medical education—embedding aging biology, healthspan extension, and lifestyle medicine at the core of training. Future physicians must be equipped not only to treat diseases, but to anticipate and prevent them by understanding the biological processes of aging. They will need to collaborate with data scientists, public health experts, and basic researchers to help shape a healthcare model that is predictive, preventive, and personalized.

The future of medicine lies in a proactive, mechanism-based approach that targets the biology of aging itself. By focusing on the shared biological underpinnings of chronic diseases, we can extend healthy longevity, improve quality of life, and reduce healthcare costs. Now, more than ever, we must invest in therapies that address aging directly—not just its consequences.

## References

[1] López-Otín C, et al. Cell. 2023; 186:243–78. 10.1016/j.cell.2022.11.001 36599349

[2] Xu M, et al. Nat Med. 2018; 24:1246–56. 10.1038/s41591-018-0092-9 29988130 PMC6082705

[3] Hickson LJ, et al. EBioMedicine. 2019; 47:446–57. 10.1016/j.ebiom.2019.08.069 31542391 PMC6796530

[4] Kirkland JL, et al. J Intern Med. 2020; 288:518–36. 10.1111/joim.13141 32686219 PMC7405395

[5] Lamming DW, et al. J Gerontol A Biol Sci Med Sci. 2020; 75:1–3. 10.1093/gerona/glz212 31544928 PMC7328347

[6] Mannick JB, et al. Nat Aging. 2023; 3:642–60. 10.1038/s43587-023-00416-y 37142830 PMC10330278

[7] Ahadi S, et al. Nat Med. 2020; 26:83–90. 10.1038/s41591-019-0719-5 31932806 PMC7301912

[8] Belsky DW, et al. Nat Aging. 2023; 3:1334–44. 10.1038/s43587-023-00518-7 37946045 PMC12459207

